# Chemical Bonding and Atomic Structure in Y_2_O_3_:ZrO_2_-SrTiO_3_ Layered Heterostructures**

**DOI:** 10.1002/ange.201108068

**Published:** 2012-02-28

**Authors:** Matthew S Dyer, George R Darling, John B Claridge, Matthew J Rosseinsky

**Affiliations:** * Department of Chemistry, University of LiverpoolLiverpool, L69 7ZD (UK)

**Keywords:** ionic conductors, metal oxides, solid-state structures

Deposition of nanosized layers of two different metal oxides upon one another produces layered heterostructures with a wide range of new physical properties.^1^ For example, a conducting two-dimensional electron gas was discovered at the interface between the two insulators LaAlO_3_ and SrTiO_3_.^2^ The atomic arrangements within heterostructures of complex functional oxides determine the resulting properties and are not necessarily straightforward to construct based on the bulk crystal structures of the independent components.

García-Barriocanal et al. reported that an epitaxial heterostructure of nanometer-sized layers of Y_2_O_3_-stabilized ZrO_2_ (YSZ) and [001]-oriented SrTiO_3_ (STO) has an up to eight orders of magnitude higher conductivity than bulk YSZ at room temperature.^3^ YSZ is a commonly used electrolyte in solid oxide fuel cells (SOFCs) run at high temperatures (>800 °C), and such an increase in ionic conductivity at lower temperatures would be expected to have a substantial impact on the performance of SOFCs.^4^ However, considerable discussion has followed the original publication, with the suggestion that the increased conductivity may be partly electronic in origin.^5^ The possibility remains that the conductivity is entirely ionic^6^ with indications that the increased conductivity arises from disorder in the oxygen lattice induced in the YSZ layers.^7^, ^8^ Similar, though smaller, increases in conductivity have been found for other layered oxide heterostructures.^9^

The exact atomic structure of the YSZ-STO heterostructures remains undetermined. Knowledge of the structure at the boundary between the two component oxide units is crucial for understanding the increase in conductivity, which has been assigned to reconstruction at this interface within the structure.^3^ It is difficult to probe the structure of interfaces within thin films experimentally, and previous attempts for YSZ-STO films seem inconsistent. The relative orientation of the YSZ and STO regions has been determined by X-ray diffraction^10^ and scanning transmission electron microscopy,^3^, ^8^ with YSZ rotated by 45° about the [001] axis relative to STO. However, electron energy loss spectroscopy suggests that the STO is terminated with a TiO_2_ layer^3^ in layered YSZ-STO, whereas experimental attempts to grow YSZ on STO substrates with different terminations show that [001]-oriented YSZ can only be reliably grown epitaxially on SrO-terminated STO,^10^ leaving the preferred termination of the STO in question. Previous calculations, carried out using only TiO_2_-terminated STO,^7^ determined that oxide ions in the first YSZ layer adopted positions completing the TiO_6_ octahedra. We assess the stability and electronic structure of different possible atomic arrangements in the YSZ/STO heterostructure using density functional theory (DFT) calculations, focussing on reconstruction at the repeating boundaries between the component YSZ and STO units. We find that the most stable structures, and those which correspond to known oxide crystal chemistry, are not obtained by taking bulk terminations of the YSZ and STO crystals. Instead taking a rock-salt ordered ZrO termination of YSZ gives stable structures with coordination environments which are consistent with known crystal structures, while maintaining insulating electronic behavior and correct YSZ stoichiometry. We have chosen to use DFT calculations, unlike recent classical force field calculations of heterostructures containing YSZ.^11^ Although DFT calculations are limited to smaller cells than those used in classical simulations, and are therefore not able to investigate the micrometer scale, they are more transferable than force fields. This is desirable for our structural investigation, since atoms in heterostructures are likely to be in coordination environments different from the constituent bulk phases. Additionally, DFT calculations allow for flexibility in the oxidation state of atoms, and for the calculation of the electronic properties of heterostructures.

The simplest model for the buried interfaces within layered heterostructures of two materials is to use terminations of their bulk crystal structures at the interfaces. The two possible terminations in the [001] direction for each of the STO (perovskite) and YSZ (fluorite) crystal structures are shown in Figure 1: STO terminates with either SrO layers or TiO_2_ layers (both neutral), and YSZ with either Zr or O_2_ layers (both charged: neglecting Y atoms and O vacancies). This yields four possible interfaces (Figure 2): A) Zr-terminated YSZ and TiO_2_-terminated STO, B) Zr-terminated YSZ and SrO-terminated STO, C) O_2_-terminated YSZ and TiO_2_-terminated STO, and D) O_2_-terminated YSZ and SrO-terminated STO.

**Figure 1:**
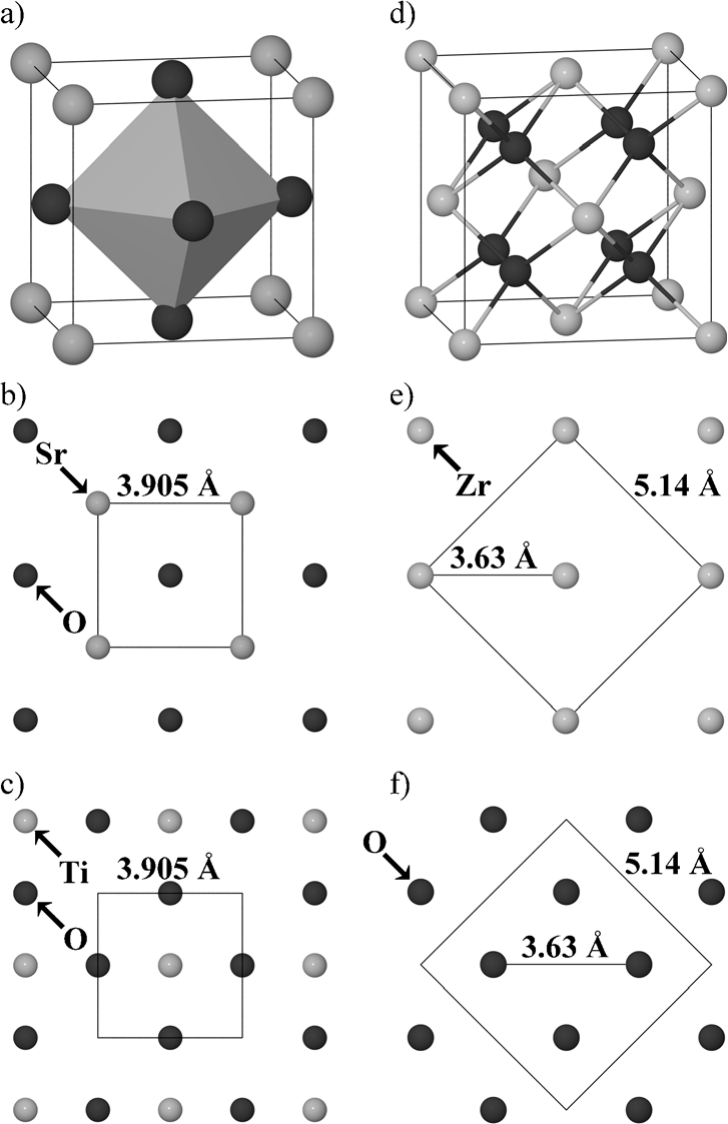
a) The perovskite crystal structure and b) the SrO- and c) TiO_2_-terminations of SrTiO_3_ in the [001] plane. d) The fluorite crystal structure and e) the Zr- and f) O-terminations of cubic ZrO_2_ in the [001] plane. The fluorite unit cell has been rotated by 45° in (e) and (f) to aid comparison with the perovskite terminations in (b) and (c) and consists of sequential charged Zr^4+^ and O_2_^4−^ layers. The ZrO termination of ZrO_2_ used to construct models **E** and **F** has the same structure as (b), where Sr is replaced with Zr. Ti atoms are at the center of the gray polyhedra, other atoms are colored as follows: Sr mid-gray, Zr light gray, and O dark gray.

**Figure 2:**
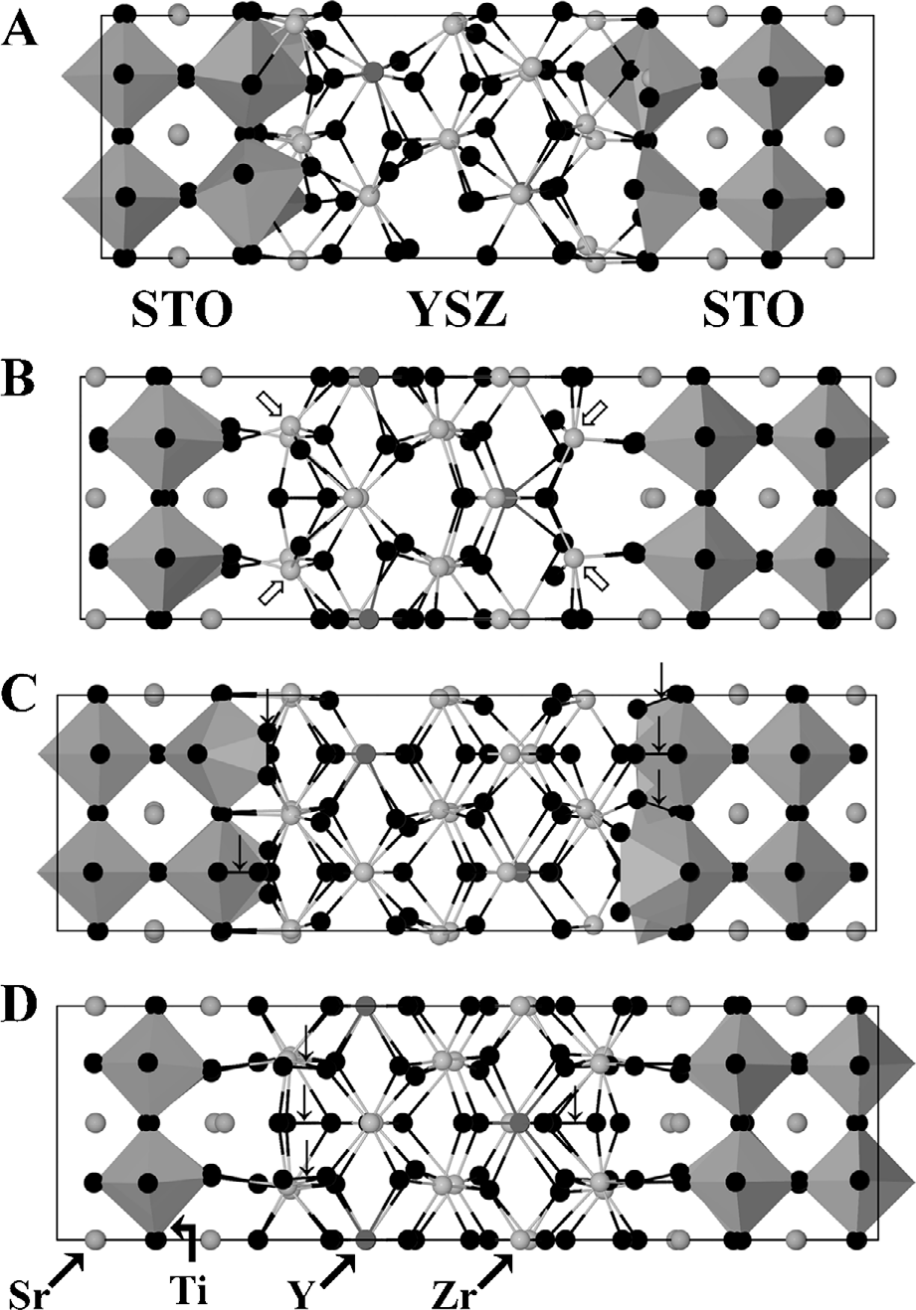
Structural diagrams of the heterostructures formed using A) Zr-terminated YSZ and TiO_2_-terminated SrTiO_3_, B) Zr-terminated YSZ and SrO-terminated SrTiO_3_, C) O_2_-terminated YSZ and TiO_2_-terminated SrTiO_3_, and D) O_2_-terminated YSZ and SrO-terminated SrTiO_3_. Each structure is viewed along one of the short lattice vectors. Ti atoms are at the center of the gray polyhedra, other atoms are colored as follows: Sr mid-gray, Zr light gray, Y dark gray, and O black. Open arrows indicate five-fold coordinated Zr atoms at the interfaces between the STO and YSZ components. Down arrows indicate O–O bonds. **A** and **C** with TiO_2_-terminated SrTiO_3_ have non-octahedrally coordinated TiO.

The construction of heterostructures containing each of these four interfaces is described in detail in the Supporting Information. We used symmetric blocks of STO and YSZ, with the same termination at each end, giving heterostructures with two equivalent interfaces. This is in agreement with experiments, which show no difference between interfaces on either side of YSZ/STO layers.^3^, ^8^ The correct choice of symmetric block is essential to obtain chemically reasonable models. The YSZ block was constructed according to the results of previous calculations.^12^ Both STO and YSZ blocks do not have the overall composition of their bulk phases, since the terminating layer is inevitably present in excess. The STO and YSZ blocks are 1–2 nm thick. Experimentally layered heterostructures have been grown with 1 nm thick YSZ layers and 10 nm thick STO.^3^ As 10 nm thick STO blocks are computationally infeasible at the level of theory used here, and the centers of the computational STO blocks already resemble bulk STO (see Table S1 in the Supporting Information), we consider our structures to be good approximations of the structures of experimental materials.

Geometry optimization of each heterostructure was performed using periodic DFT,^13^ and as an indication of the relative stability of each heterostructure, a heat of formation was calculated with respect to the binary oxides, as described in the Supporting Information. The lowest energy structure for each of the four heterostructures, **A**–**D**, is shown in Figure 2. The calculated energies were 26.3, 27.1, −0.5, and −5.1 eV for **A**–**D**, respectively, with lower values representing more stable structures.

Structures **A** and **B**, with Zr-terminated YSZ blocks, are clearly unstable. The Zr(Y) and O_2_ layers of YSZ are formally charged, whereas the SrO and TiO_2_ layers of STO are charge neutral. As a result, the symmetric blocks of YSZ built with bulk terminations are formally charged. With Zr-terminated blocks, the additional layer of Zr atoms gives a composition of [Y_2_O_3_][ZrO_2_]_14_Zr_4_ for the YSZ block, with four excess Zr^4+^ ions. The calculations impose charge neutrality within the super-cells, which therefore have more electrons than expected for the ions Sr^2+^, Ti^4+^, Y^3+^, Zr^4+^, and O^2−^. Plots of the partial density of states (PDOS) of **A** and **B** (Figure S2 in the Supporting Information) show that the excess electrons populate Zr states within the band gap, and begin to fill the Ti-dominated conduction band. This reduction of Zr^4+^ and Ti^4+^ to Zr^3+^ and Ti^3+^ is clearly energetically expensive, leading to the instability of these models.

The interfaces within **A** and **B** also suggest energetically unstable structures. The Ti octahedra terminating the STO block in **A** (Figure 2 A) are clearly distorted, in some the Ti atom is only five-fold coordinated, in a square-pyramidal geometry. The Zr atoms at the interface in **B** are similarly under-coordinated with most only bonded to five O atoms (Figure 2 B). To our knowledge five-coordinate Zr is unknown in oxide crystal structures.

In contrast, structures **C** and **D**, are constructed using O_2_-terminated YSZ blocks. These structures represent most closely those proposed in earlier experimental studies. García-Barriocanal et al.^3^ proposed TiO_2_-terminated STO and O_2_-terminated YSZ, similar to structure **C**. Structure **D**, with SrO-terminated STO and O_2_-terminated YSZ, is effectively that proposed by Cavallaro et al.^10^ with an interface region resembling SrZrO_3_. Both models have YSZ blocks with composition [Y_2_O_3_][ZrO_2_]_18_O_8_. Considering the expected oxidation states of all the ions, the eight excess O atoms result in electron deficient charge-neutral super-cells. However the plots of the partial density of states (PDOS, Figure S2 in the Supporting Information) do not show unoccupied states at the top of the valence band as might be expected. Instead O–O bonding is present in the relaxed structures of **C** and **D**. Structure **C** contains eight O–O bonds of lengths 1.4–1.5 Å in the interface region, causing over coordination of Ti atoms, evident in Figure 2. This O–O bonding completely accounts for the electron deficiency in the super-cell. Similarly, structure **D** contains six O–O bonds of 1.5 Å length, with an extra O in the Zr layers closest to the interface. Again, this largely accounts for the electron deficiency in the super-cell, however with only six and not eight O–O bonds, the valence band is now partially occupied (Figure S2 in the Supporting Information). No other structure presented herein contained O–O distances shorter than 2 Å.

Using symmetrical unreconstructed Zr- and O_2_-terminated YSZ blocks clearly leads to heterostructures which do not follow conventional solid-state chemistry. They are either reduced in the case of Zr excess, or contain O–O bonds in the case of O excess. It is, however, possible to construct symmetric YSZ blocks which are formally charge neutral. Adding O to each end of Zr-terminated YSZ blocks (Figure 1 e), gives rock-salt ordered ZrO terminating layers, similar to the SrO layers in STO (Figure 1 b, and Figure S3 in the Supporting Information) and as observed experimentally for the surface of YSZ.^14^ The resulting symmetric ZrO-terminated YSZ blocks have the composition [Y_2_O_3_][ZrO_2_]_18_, and thus have neither excess Zr nor excess O. The ZrO-terminated YSZ blocks were combined with TiO_2_- and SrO-terminated STO blocks giving structures **E** and **F**, respectively. The final structures, optimized as for **A**–**D**, are shown in Figure 3.

**Figure 3:**
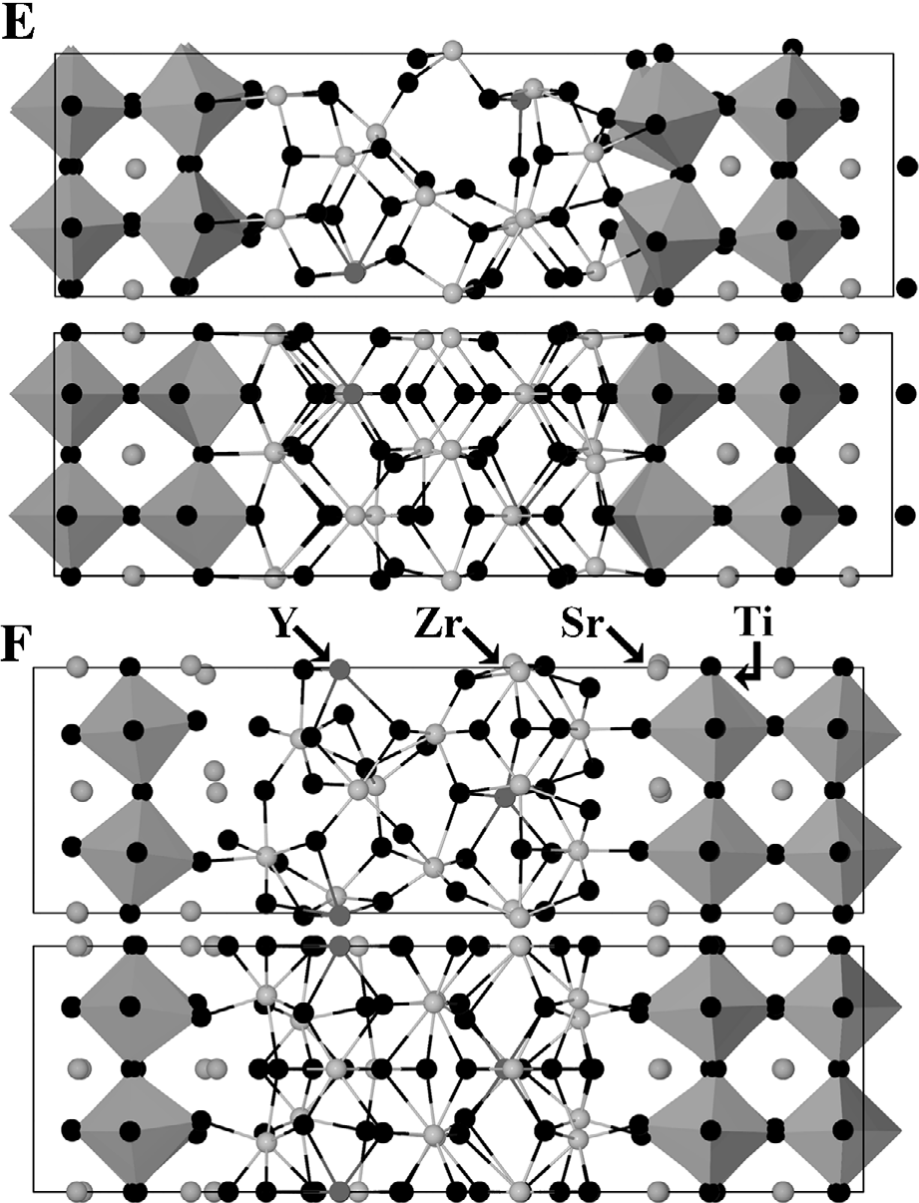
Structural diagrams of the interfaces formed within an STO-YSZ heterostructure using a ZrO-terminated YSZ block (depicted in Figure S3 in the Supporting Information) and **E**: TiO_2_-terminated and **F**: SrO-terminated SrTiO_3_. To best display these two important structures, each structure is viewed along both of the two short lattice vectors, rather than just one. Ti atoms are at the center of the gray polyhedra, other atoms are colored as follows: Sr mid-gray, Zr light gray, Y dark gray, and O black.

The calculated heats of formation for **E** and **F** were −2.5 and −6.7 eV, respectively. Structure **E** is more stable than the other two heterostructures with TiO_2_-terminated STO (**A**: 26.3 eV and **C**: −0.5 eV), and structure **F** is more stable than those with SrO-terminated STO (**B**: 27.1 eV and **D**: −5.1 eV). The heterostructure with SrO-terminated STO, **F**, is more stable than **E**, in good agreement with experimental observations that epitaxial growth of [001] oriented YSZ was only seen with SrO-terminated STO substrates.^10^

Models **E** and **F** show none of the structural problems found in **A**–**D**. The PDOS plots in Figure 4 show that both are undoped semiconductors, and no O–O bonds are present in their structures. The calculated gaps (**E**: 1.78 eV, **F**: 1.37 eV) are similar to the calculated gap of STO (1.79 eV) which is smaller than that of YSZ. We therefore consider **E** and **F** to be the best representative models of YSZ-STO heterostructures proposed to date. We emphasise that they require reconstruction of the terminal layer of the YSZ block away from the bulk termination.

**Figure 4:**
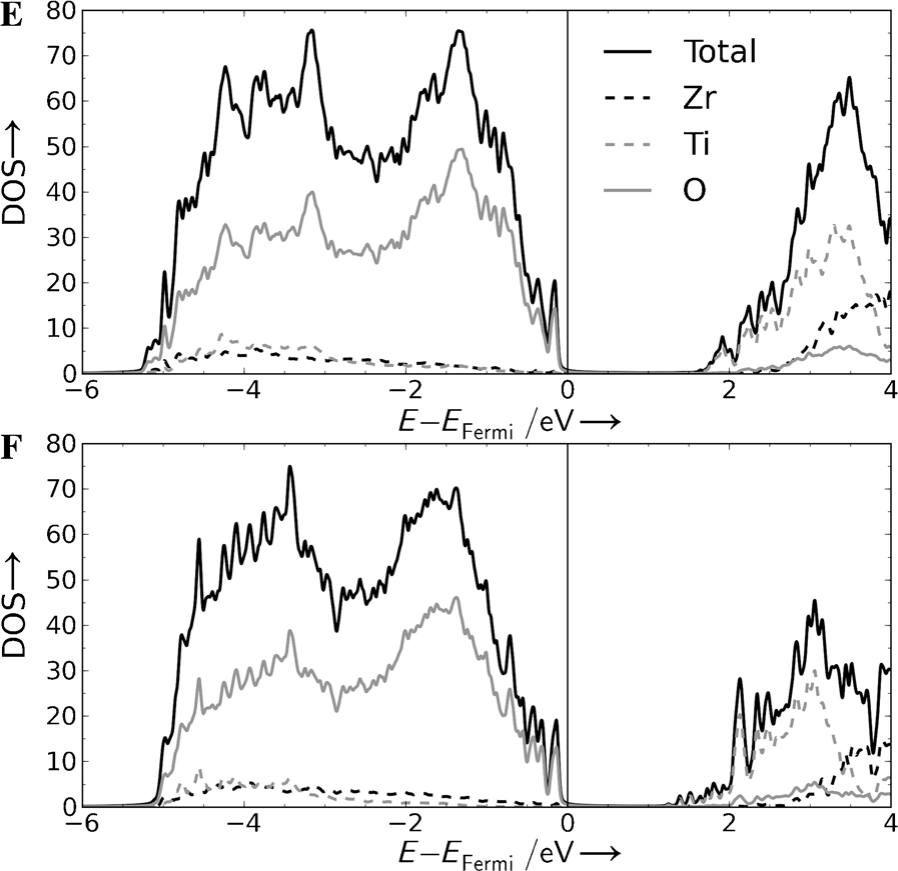
Partial density of states of structures formed with ZrO-terminated YSZ and **E**) TiO_2_-terminated and **F**) SrO-terminated SrTiO_3_. Both materials have significant band gaps (DOS=density of states).

A closer look at the structures of **E** and **F** in Figure 3 shows a surprising level of order at the interfaces between YSZ and STO, particularly the left-hand interface of **E**, and the right-hand interface of **F**. The first layers of YSZ at these two interfaces appear well ordered, although the other interfaces are less so. Furthermore, all of the Zr atoms in the YSZ layers directly neighboring the interfaces are seven-fold coordinate. This is the preferred coordination number of Zr in baddeleyite (monoclinic ZrO_2_),^15^ and an entirely reasonable coordination environment for the Zr atoms. In agreement with earlier work,^7^ we find that the Ti atoms in **E** are octahedrally coordinated. The YSZ terminating ZrO layer effectively caps the TiO_2_-terminating layer of STO continuing the perovskite structure with Zr in the A-site. In **F**, the terminating ZrO layer lies above the SrO-terminating layer of STO in a rock-salt like geometry resembling the layers of neighboring perovskite blocks in Ruddlesden–Popper structures.^16^ Thus, in both **E** and **F**, the ordered interfaces can be described in terms of simple, well-known structural motifs for metal oxides.

The central region of YSZ in both **E** and **F** appears considerably less ordered than at the interfaces. This disorder is largely due to the strain imposed on the YSZ by the STO substrate, consistent with previous work.^7^, ^8^ Comparison of bulk YSZ structures calculated with and without strain in Figure S1 in the Supporting Information, clearly shows the deviation from the fluorite structure upon the application of strain. One indication of this structural change is the alteration in the number of Zr and Y atoms bonded to each O. In unstrained YSZ this is four for each O atom, as expected in the fluorite structure. With the application of strain this drops to an average of 3.7 for bulk YSZ, similar to the values of 3.6 and 3.9 for the YSZ regions of **E** and **F** (neglecting O atoms at the interface). Similarly the average number of O atoms bonded to Zr and Y atoms drops from 7.8 in unstrained YSZ to 7.2 in strained YSZ and 7.1 and 7.3 in **E** and **F**. Although it is tempting to relate this change away from the fluorite structure to the increased ionic conductivity seen experimentally, we note that the O and Zr environments are in fact becoming close to those found in the most stable ZrO_2_ polymorph, baddeleyite. Baddeleyite has a distorted fluorite structure with an average of 3.5 Zr atoms bonded to each O atom, and each Zr atom coordinated to 7 O atoms,^15^ and YSZ in this structure does not have a greater ionic conductivity than cubic YSZ.^17^ Previous calculations on strained YSZ do suggest enhanced O diffusion, however not sufficient to fully account for the increased ionic conductivity observed in YSZ-STO heterostructures.^18^ In contrast to the YSZ region, the central STO region in all models remains largely unchanged (Table S1 in the Supporting Information), retaining the perovskite structure.

The results of this study suggest that the buried YSZ-STO [001] interfaces within the heterostructure require reconstruction during the growth process of the component structures to permit energetically stable and crystal-chemically acceptable structures to form. We also confirm that the most stable heterostructure is formed with YSZ and SrO-terminated STO, in agreement with experiment.^10^ The structures proposed here can be used in calculating properties relevant to ionic transport, such as the influence of oxygen defects, or the concentration and site preference of Y atoms (in particular their proximity to the YSZ-STO interface), or molecular dynamics simulations. The prediction of possible structures could also be an aid to further experimental characterization of YSZ-STO heterostructures. It is interesting to note the relative stability of structures containing excess oxygen at the interface (models **C** and **D**), compared to those with oxygen deficiency (models **A** and **B**). This suggests further research into the possible contribution of excess oxygen at YSZ-STO interfaces to increased ionic conductivity.

This study shows the benefit of choosing alternative terminations to the simple bulk terminations of materials when constructing repeating buried interfaces in artificial heterostructures. This is consistent with the known reconstruction of oxide surfaces, including YSZ[001]^14^, ^19^ and STO[001]^20^ but distinct as in a heterostructure the interfaces are buried. Our findings are relevant for such buried interfaces in many heterostructures, particularly those with layers which are not charge neutral such as A(III)B(III)O_3_ perovskites and materials with the fluorite structure.

## Supplementary material

Detailed facts of importance to specialist readers are published as ”Supporting Information”. Such documents are peer-reviewed, but not copy-edited or typeset. They are made available as submitted by the authors.
