# Supplementary material for: Chemical Bonding and Atomic Structure in Y2O3:ZrO2-SrTiO3 Layered Heterostructures
Source: Angew Chem Weinheim Bergstr Ger. 2012 Feb 28;124(14):3474–8. doi: 10.1002/ange.201108068 (PMC4373140; doi:10.1002/ange.201108068)
Supplement: Supplementary file 1 [file ange0124-3474-SD1.pdf]

Angewandte Chemie
Eine Zeitschrift der Gesellschaft Deutscher Chemiker

Supporting Information

© Wiley-VCH 2012

69451 Weinheim, Germany

Chemical Bonding and Atomic Structure in Y_2O_3:ZrO_2-$SrTiO_3$ Layered Heterostructures**

*Matthew S. Dyer, George R. Darling, John B. Claridge, and Matthew J. Rosseinsky**

ange_201108068_sm_miscellaneous_information.pdf

Supporting Information

Table of Contents

Full author list for reference [5a]
Computational Details
SI Figure 1: Structural diagrams of bulk YSZ
SI Figure 2: Partial density of states plots
SI Figure 3: Reconstructed YSZ block
SI Table 1: Ti–O bond lengths from the centre of STO blocks
Structure files: VASP geometry files of all heterostructures

[5] a) A. Cavallaro, M. Burriel, J. Roqueta, A. Apostolidis, A. Bernardi, A. Tarancón, R. Srinivasan, S. N. Cook, H. L. Fraser, J. A. Kilner, D. W. McComb, J. Santiso, *Solid State Ionics* **2010**, *181*, 592

Computational Details

As discussed in the main text, there are four possible combinations of STO and YSZ blocks when using terminations from the bulk crystal structures: (**A**) Zr-terminated YSZ and TiO_2-terminated STO, (**B**) Zr-terminated YSZ and SrO-terminated STO, (**C**) O_2-terminated YSZ and TiO_2-terminated STO, and (**D**) O_2-terminated YSZ and SrO-terminated STO. By using symmetric blocks of STO and YSZ, which are terminated by the same layer on each side, we construct heterostructures which contain only one of the four combinations, **A**–**D**. The relative lateral placement of the STO and YSZ blocks in each case is unknown, so for each of the four combinations, **A**–**D**, we have constructed two model heterostructures, which differ only by the relative lateral positions of the STO and YSZ blocks. The YSZ block of one model was displaced by (1/4,1/4,0) relative to that of the second, with the STO block in the same position. The most stable of these two structures is then used in our discussion of the structures and relative stabilities of the heterostructures labelled **A**–**D**.

The initial STO blocks consisted of seven layers, terminated with either SrO or TiO_2, with the calculated equilibrium lattice parameter of 3.94 Å. They have compositions of $[SrTiO_3]_{12}[SrO]_4$ and $[SrTiO_3]_{12}[TiO_2]_4$ respectively. The YSZ blocks were constructed using a $\sqrt{2}\times\sqrt{2}\times 2$ fluorite super-cell of ZrO_2, replacing two Zr atoms with Y and removing an O atom next-nearest-neighbor to both Y atoms.[12] The resulting $[Y_2O_3][ZrO_2]_{14}$ super-cell was relaxed twice, once keeping the in-plane cell parameters at 7.88 Å and once relaxing the full cell, as described below. The final structures are shown in SI Figure **1**. The symmetric blocks used to build the YSZ-STO interfaces were then created by adding a Zr layer to one side of the strained structure, to create a nine layer Zr-terminated YSZ block with composition $[Y_2O_3][ZrO_2]_{14}Zr_4$ (used in **A** and **B**), then adding two further O_2 layers creating an eleven layer O_2-terminated YSZ block with composition $[Y_2O_3][ZrO_2]_{18}O_8$ (used in **C** and **D**). The YSZ blocks were rotated by 45° about the [001] direction relative to STO blocks as shown in Figure **1**, in agreement with experiment.[3,10]

The blocks are constructed with one Y atom in the second layer of cations away from each interface, as can be seen in Figure **2**. It is known that Y atom segregation occurs at the surface of YSZ[M. de Ridder, R. G. van Welzenis, A. W. Denier van der Gon, H. H. Brongersma, S. Wulff, W.-F. Chu, W. Weppner, *Journal of Applied Physics* **2002** *92* 3056; H. B. Lee, F. B. Prinz, W. Cai, *Acta Materialia* **2010** *58* 2197], so further studies in which the preferred location of the Y atoms within the YSZ layer, although beyond the scope of the current work, would be of interest.

Geometry optimizations were carried out using periodic DFT with the PBE functional, with the VASP code. Interactions with core electrons were treated using the projector augmented wave method.[12] The number of plane-waves was limited by a 500 eV energy cutoff, and a 6×6×2 *k*-point grid was used. To model the strain due to thin-film growth on an underlying STO substrate, the in-plane cell parameters of all models were constrained to 7.88 Å. The out-of-plane cell parameter was varied by hand, with the forces on each atom minimized to below 0.01 eV/Å at each point, until a minimum energy structure was found.

Heats of formation at 0 K were calculated for each structure relative to the binary oxides SrO, TiO_2, Y_2O_3 and TiO_2. Where necessary the calculation included the energy for gas phase O_2 to compensate for oxygen excess or deficiency. For example the heats of formation, $\mathit{\Delta}H_F$, of **A** and **D** were calculated as follows:

$$\mathit{\Delta}H_F(\mathbf{A}) = E(\mathbf{A}\text{:}[SrTiO_3]_{12}[TiO_2]_4[Y_2O_3][ZrO_2]_{14}Zr_4) - 12E(SrO) - 16E(TiO_2) - E(Y_2O_3) - 18\,E(ZrO_2) + 4E(O_2)$$
$$\mathit{\Delta}H_F(\mathbf{D}) = E(\mathbf{D}\text{:}[SrTiO_3]_{12}[SrO]_4[Y_2O_3][ZrO_2]_{18}O_8) - 16E(SrO) - 12E(TiO_2) - E(Y_2O_3) - 18\,E(ZrO_2) - 4E(O_2)$$

The heats of formation allow us to compare the stability of heterostructures with different composition, but we note that we are not attempting to find the most stable composition in the Sr-Ti-Y-Zr-O phase diagram. Structures grown by layered deposition techniques are likely to be kinetically trapped and not at thermodynamic equilibrium.

The relative heats of formation are equivalent to calculating the relative energies of each model at a constant composition of $[SrTiO_3]_{16}[Y_2O_3][ZrO_2]_{18}$, where SrO, TiO_2 and gas phase O_2 are used to bring each model to this composition. Again, we use the examples of **A** and **D**:

$$E_{relative}(\mathbf{A}\text{: }[SrTiO_3]_{16}[Y_2O_3][ZrO_2]_{18}) = E(\mathbf{A}\text{:}[SrTiO_3]_{12}[TiO_2]_4[Y_2O_3][ZrO_2]_{14}Zr_4) + 4E(SrO) + 4E(O_2)$$
$$E_{relative}(\mathbf{D}\text{: }[SrTiO_3]_{16}[Y_2O_3][ZrO_2]_{18}) = E(\mathbf{D}\text{:}[SrTiO_3]_{12}[SrO]_4[Y_2O_3][ZrO_2]_{18}O_8) + 4E(TiO_2) - 4E(O_2)$$
$$\mathit{\Delta}E_{relative} = E_{relative}(\mathbf{D}) - E_{relative}(\mathbf{A}) = E(\mathbf{D}) - E(\mathbf{A}) + 4E(TiO_2) - 4E(SrO) - 8E(O_2) = \mathit{\Delta}H_F(\mathbf{D}) - \mathit{\Delta}H_F(\mathbf{A})$$

This is close to experimental conditions in which STO and YSZ are depositted in a known composition, and any deviation away from this composition in the YSZ-STO heterostructures is likely to be balanced by the presence of binary oxides of the excess material elsewhere in the sample or a change in oxygen content.

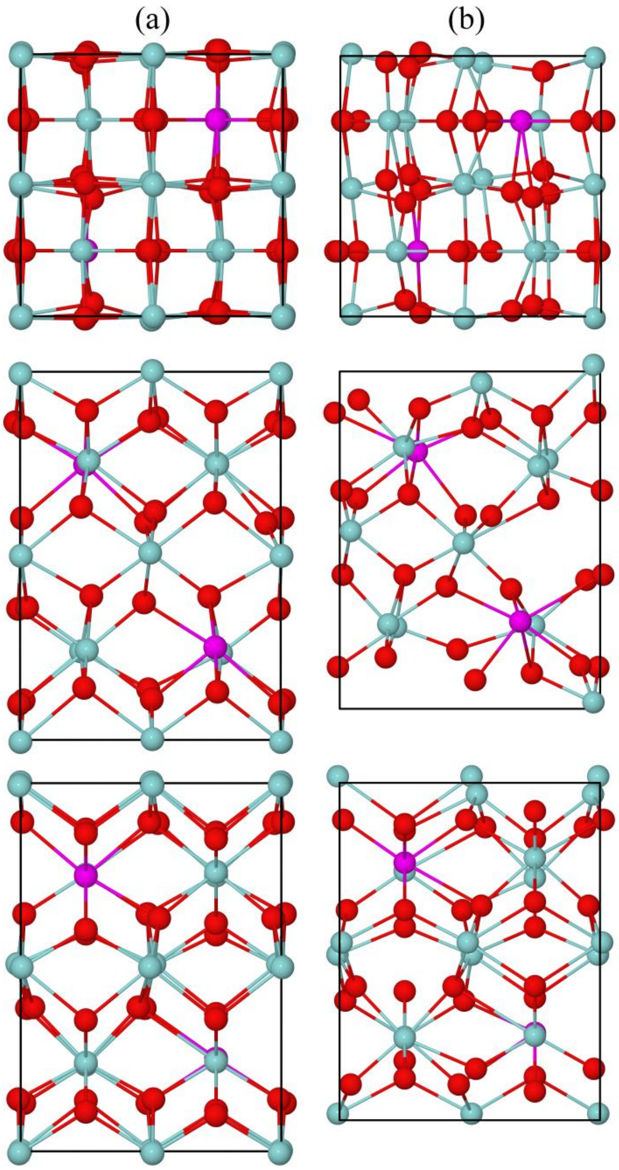

SI Figure 1: Structure of YSZ, (a) unstrained and (b) strained in the ab-plane to the $SrTiO_3$ lattice vectors. Structures are shown along each of the three lattice directions. Atoms are colored as follows: Y pink, Zr light blue, O red. Unstrained YSZ (a) retains the cubic fluorite structure of cubic ZrO_2 with some distortion of bond lengths and angles due to the oxygen vacancy and Y substitutions. When put under strain to match $SrTiO_3$ in the ab-plane, (b), considerable rearrangement occurs. The structure no longer looks like the fluorite structure, with many Y and Zr atoms coordinated to seven oxygen atoms. The strained model was used as the starting point for the construction of YSZ blocks in all of the heterostructures.

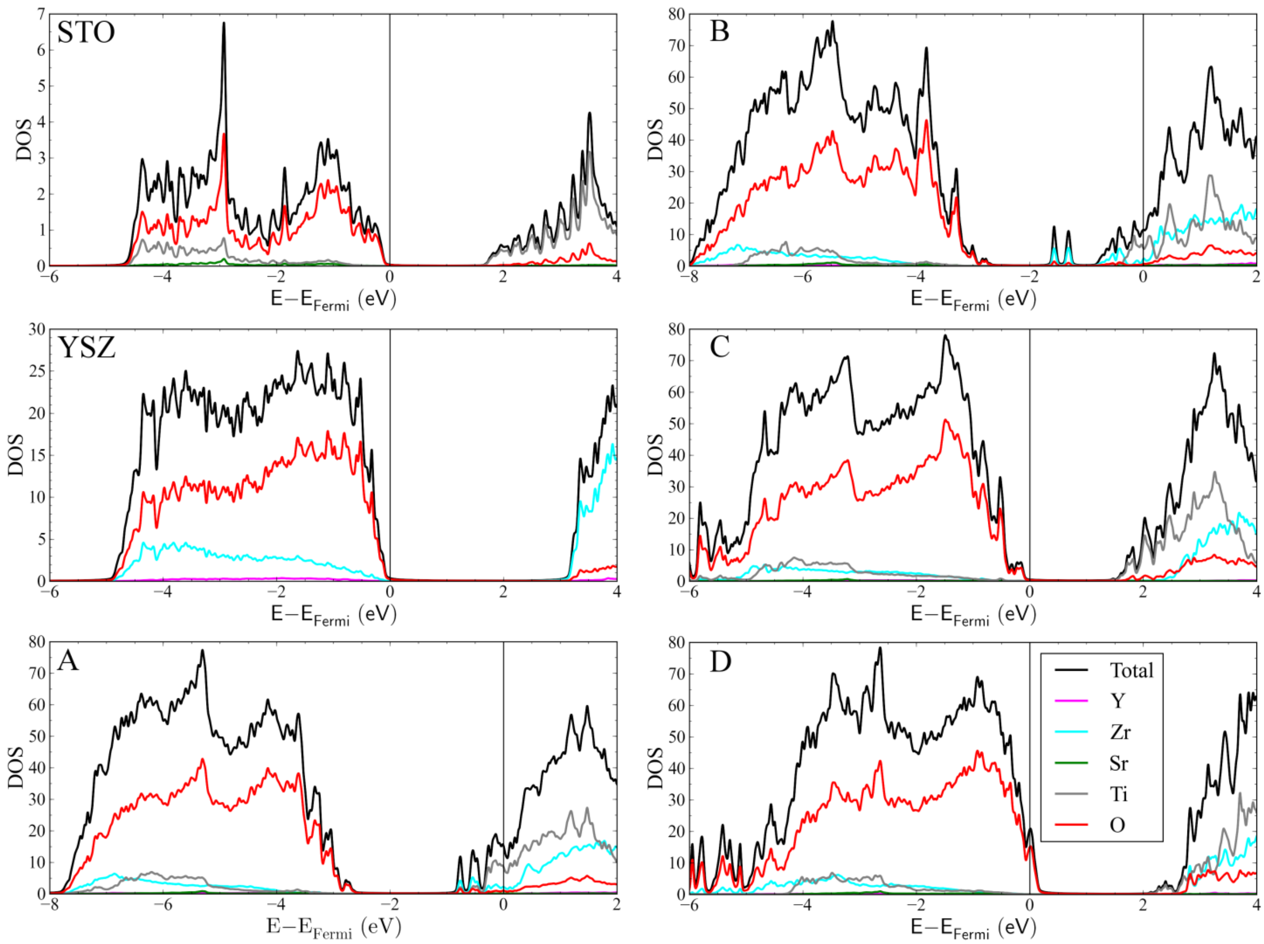

SI Figure 2: Partial density of states of STO: $SrTiO_3$, YSZ: strained Y_2O_3 stabilized ZrO_2, **A**: Zr-terminated YSZ and TiO_2-terminated $SrTiO_3$, **B**: Zr-terminated YSZ and SrO-terminated $SrTiO_3$, **C**: O-terminated YSZ and TiO_2-terminated $SrTiO_3$, and **D**: O-terminated YSZ and SrO-terminated $SrTiO_3$.

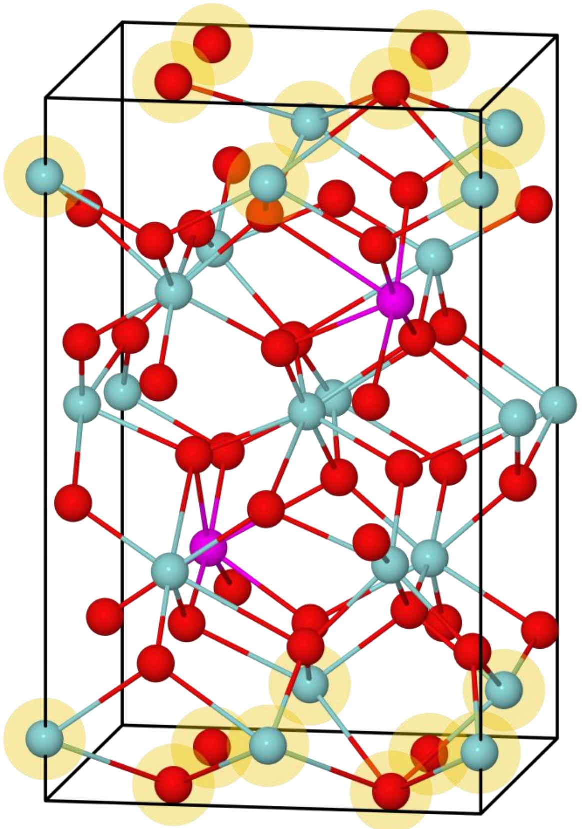

SI Figure 3: The reconstructed YSZ block used to construct **E** and **F**. The terminations at each end of the block (atoms highlighted in yellow) have four Zr and four O atoms in a rumpled rock-salt formation.

Structure	Ti–O (equatorial) / Å	Ti–O (axial) / Å
$SrTiO_3$ (experimental)	1.9525	1.9525
$SrTiO_3$ (calculated)	1.97	1.97
**A**	1.972 ± 0.009	2.04 ± 0.09
**B**	1.972 ± 0.004	2.037 ± 0.008
**C**	1.97 ± 0.04	2.0 ± 0.1
**D**	1.971 ± 0.005	1.95 ± 0.01
**E**	1.97 ± 0.05	2.0 ± 0.1
**F**	1.98 ± 0.01	2.0 ± 0.2

SI Table 1: Mean equatorial and axial bond Ti–O lengths taken from the central Ti layer(s) in the relaxed heterostructures, and a comparison to data for cubic $SrTiO_3$. The single central layer was used for SrO terminated $SrTiO_3$ blocks, and the two central layers for TiO_2 terminated $SrTiO_3$ blocks. In all cases the central layer retained the cubic perovskite structure, with distortions of the axial bonds of <10% on average.

Structure files

Structure files for models **A**–**F** are attached in the VASP POSCAR file format. A brief summary of the format is:

Line 1: Comment line (in these files contains list of atomic species as in line 6)
Line 2: Scale factor for cell vectors and positions (in these files no scaling is done)
Lines 3-5: Unit cell vectors in Cartesian coordinates and Å
Line 6: List of atomic species in order
Line 7: Number of atoms of each species in order
Line 8: Direct/Cartesian coordinates (these files use direct)
Lines 9-end: Positions of each atom as fraction of lattice vectors

Model A

```
 O Sr Ti  Y Zr
 1.00000000000000000
     7.8799999999999999    0.0000000000000000    0.0000000000000000
     0.0000000000000000    7.8799999999999999    0.0000000000000000
     0.0000000000000000    0.0000000000000000   25.0899999999999999
   O    Sr   Ti   Y    Zr
  75  12  16   2  18
Direct
  0.9744972017916587  0.7160638332216835  0.0551222996277021
  0.2904727784655741  0.8993193302096993  0.9717362801392007
  0.1220410266149506  0.2262645183335210  0.9779942136225588
  0.1834312532790930  0.0263924237645339  0.8694565515571671
  0.7748028141277887  0.0174776525283203  0.9516281982840623
  0.9989160714569566  0.7410529129865768  0.9492093552548562
  0.4928172621019845  0.2344168837839437  0.9338277762403465
  0.3097568225946423  0.4435043172690409  0.8466887444190740
  0.7478526194872249  0.0384226170242763  0.8517228016121763
  0.0476746955569359  0.7264771199747363  0.8451252752377996
  0.2730078855488648  0.5265448302862726  0.9465804449946138
  0.8008080730417843  0.5275267647423788  0.6615384914829534
  0.9750293460153893  0.7582968782902605  0.2214966211083605
  0.6953897927080039  0.7369142287548600  0.4628136923886957
  0.7331438920083242  0.7586551302694244  0.1402902408650764
  0.7280519817364293  0.5032954823146292  0.2210215003944562
  0.9941420297044752  0.3556867834694200  0.8777503630705301
  0.4755740194164575  0.7526472903965330  0.2224455638335294
  0.7796220688087915  0.3769908796134452  0.9786347364819118
  0.4807737828489443  0.8116494001667302  0.8634735445412512
  0.6145170761689408  0.7155012463712351  0.9751298456341332
  0.5663007110389566  0.2945426363582291  0.6591432549797953
  0.0274724033238084  0.7124374600551109  0.7441277244484392
  0.3141743616157536  0.5375510582066221  0.7525448264836150
  0.0631474870780746  0.7683798880558323  0.6343483813438168
  0.2902252253386544  0.5260299873942175  0.6503304290766884
  0.3063012910061479  0.0159404489121384  0.6531216131230823
  0.5319594298022908  0.2355654480318248  0.7677034624480905
  0.8076473507464602  0.0113126365679030  0.7504439761294831
  0.0254513620571829  0.2799816974193591  0.6699873169070880
  0.3145004302978265  0.9249990876457103  0.7649878280430412
  0.1121793752003866  0.2296932303855036  0.7744429366432533
  0.6511275655487930  0.7237543714628711  0.7723119086130203
  0.7891018071129199  0.0294137235060796  0.6487939762905318
  0.6607950962865076  0.4861900160115631  0.8737764312964366
  0.8158635539421674  0.4196751558336165  0.7721066757814428
  0.7878247563264610  0.0160650398562489  0.0552104917708861
  0.4695775489870609  0.1693049786424392  0.5494052608033522
  0.2246381960574489  0.7407369561698627  0.1396127910713824
  0.2251238135936531  0.9966223090181582  0.3816663665531221
  0.7490242688264257  0.9845407161069584  0.5414129794765367
  0.9629391973213979  0.6618397216220872  0.5408334726461488
  0.2119719037714883  0.2405915064312252  0.4623079142932405
  0.1536615996478393  0.9919689979754326  0.5443138686757042
  0.9484182248987386  0.2833841216546608  0.5600529010779590
  0.7260952474335719  0.2480732366440363  0.4640493606437666
  0.7302660030860150  0.7504367532617503  0.3021267039875251
  0.7254487832855591  0.4962811103236874  0.3810255963737004
  0.4712209069421634  0.7450477355086603  0.3789500717912870
  0.4840537389976419  0.8502755577730666  0.0647829603915074
```

0.2167730686959695	0.7546866503486955	0.3015355430566957
0.2228269021813626	0.2455791052423264	0.3007073383562073
0.2208349882955657	0.4971218619735123	0.3808382279289626
0.9728562079416980	0.7482549499310767	0.3865809264832954
0.2453217994135842	0.4838539928167420	0.5434501398640141
0.2355003776010502	0.7461188351874245	0.4653824486286346
0.7259063285093622	0.2493250153430529	0.3010066151991756
0.7211587008745342	0.9969417676780565	0.3826006211420545
0.4734185144332105	0.2481173589976106	0.3823063565882785
0.4504582920674557	0.7736506055317783	0.5634804324601205
0.6486101450360050	0.4852470467876897	0.5485795001677544
0.1631633355878926	0.0139245114346276	0.0672402911584081
0.9762271606557802	0.2520807900903573	0.2182838916875015
0.2489338562698033	0.2704812125705345	0.1370939328103845
0.2221602613582611	0.5039766635299011	0.2233508715735803
0.4848288246952366	0.2209493780631750	0.0411304083279004
0.9721888607266689	0.2439967258560606	0.3806857952660221
0.6437925139591615	0.5114772601734208	0.0643989985878376
0.7188072345022197	0.2485480588912688	0.1378993562067444
0.2274334308100368	0.0039935792904272	0.2181225877310685
0.5368513942757731	0.7766793275403766	0.6734755153861980
0.7226786266806344	0.0041882418440923	0.2220096497474333
0.4761813405514313	0.2571521886984097	0.2225432607234719
0.9833184712824693	0.3471415253673548	0.0660384335459321
0.2903531634725160	0.5166736883275799	0.0483025843176050
0.4741757811851967	0.0005427815999202	0.3004902923364526
0.9781993808975823	0.5117330987376033	0.1509234009240123
0.9748449824643234	0.5002217591166217	0.3005065223523028
0.9740400552685965	0.0011961761895551	0.3002345362367819
0.9772914279062634	0.0009172304491551	0.4531763664764789
0.4736898450489803	0.5006562177803872	0.3005097499774543
0.9709941709173986	0.0013823546373496	0.1491082061510045
0.4719974990340234	0.5000342953153858	0.4530834040228444
0.9792264064058425	0.4928726057479624	0.4521171039415319
0.4761002164887301	0.9938002796423857	0.4519743918829420
0.4760535575462575	0.5066437481182385	0.1498216948044957
0.4770571204728922	0.0072698455745936	0.1507102225115716
0.2430875643530857	0.2425739966879986	0.5379168646985142
0.7451309642078189	0.2652443128889966	0.0636474857822339
0.7379094240676856	0.7406839421888111	0.5371926516833754
0.2265413089749988	0.2544004311054033	0.2222468667339751
0.7251315052319769	0.2534753394638916	0.2222344322932560
0.7266008349936836	0.7503459072891512	0.0638165320708708
0.2412778724271226	0.7636412202357440	0.0637994439961238
0.2319413243432191	0.2579923408755699	0.0620938991711855
0.2244795643708304	0.7536232257657610	0.2219310237620166
0.2232861229075658	0.2464070749672627	0.3786910905520015
0.7211546275193600	0.2405187572447609	0.5369141907307542
0.2128494805516511	0.7461753184996173	0.5392349932796885
0.7240946566408812	0.2481968835115062	0.3774018851303619
0.7260004102498439	0.7540168952615440	0.2225787510573011
0.2233248172620746	0.7486684614385949	0.3779634919546880
0.7227488737500715	0.7469111142704082	0.3790991712923034
0.3056364435714420	0.2707746220293846	0.7045384942392235
0.7638583982337775	0.7676215633340296	0.8976881288388393
0.7631353768519366	0.2534473036112907	0.9034112636927033
0.0474439587938703	0.4874742548572691	0.6100903051141455
0.5498348721273689	0.9804090393809471	0.6102680297037169
0.2476603977670814	0.7646776979900871	0.9005466996405848
0.8217033736238152	0.7884684726379597	0.6923416503881489

```
  0.7806087319625677  0.2628739318102700  0.7077716991960208
  0.5534006784530976  0.5525841945492694  0.6226866511520025
  0.5452828346915592  0.4923400884928559  0.8002265412100853
  0.2570684488223486  0.7684321108502030  0.6968054831562641
  0.2388967961899213  0.2605599116408164  0.9025036983758312
  0.0613961220260712  0.9727155418073362  0.7899291655933717
  0.0755295631164017  0.4878379622514167  0.7991578568379659
  0.0120426190182589  0.9643920813108313  0.9997200098674655
  0.5393285996911443  0.9911035279901118  0.9955090902699594
  0.5132560117505683  0.4732328795227967  0.9907792383289648
  0.5688545407224506  0.9616474241956829  0.7952269805166288
  0.0437909760457131  0.5036722313920717  0.9982329029210252
  0.0512430691666651  0.0516930936187573  0.6209023055693662
```

Model B

```
 O Sr Ti  Y Zr
 1.0000000000000000
     7.8799999999999999    0.0000000000000000    0.0000000000000000
     0.0000000000000000    7.8799999999999999    0.0000000000000000
     0.0000000000000000    0.0000000000000000   25.7199999999999989
   O     Sr    Ti    Y     Zr
  71  16  12   2  18
Direct
  0.2327067267182352  0.7300369426353323  0.2258676762100036
  0.2133695796360978  0.5000000000000000  0.9021986076595599
  0.4776876536461611  0.2396711849423549  0.9422245180357030
  0.3218925564097539  0.5000000000000000  0.9994115902565157
  0.4904411591611422  0.3131204980031210  0.8401841611828555
  0.9868089432361123  0.3012571999683304  0.9713318114722292
  0.2185468402283830  0.0000000000000000  0.9461447313322100
  0.9868089432361123  0.6987428000316624  0.9713318114722292
  0.7055177799087551  0.5000000000000000  0.9377188158981210
  0.9034044459482076  0.2807293194131535  0.8627153678312552
  0.2665385527331317  0.0000000000000000  0.8424905300253595
  0.4776876536461611  0.7603288150576450  0.9422245180357030
  0.6089598342319987  0.0000000000000000  0.8279359809162304
  0.4884105986854780  0.2529691102090730  0.1501122405727171
  0.2430873343331466  0.2097558535440749  0.0607556715728074
  0.4904411591611422  0.6868795019967582  0.8401841611828555
  0.4884105986854780  0.7470308897909270  0.1501122405727171
  0.9238007406531308  0.0000000000000000  0.8017236275464042
  0.5193879261747401  0.2240682883137781  0.7415191939347878
  0.9034044459482076  0.7192706805867328  0.8627153678312552
  0.0236504002371094  0.1774334103943508  0.6498084382699247
  0.6929498919066639  0.0000000000000000  0.6140929671775769
  0.0236504002371094  0.8225665896056492  0.6498084382699247
  0.7369733170271462  0.0000000000000000  0.9227435041860473
  0.8261702141140659  0.5000000000000000  0.6622354716864639
  0.2877497591000658  0.5000000000000000  0.7628295005457940
  0.2695664681019984  0.0000000000000000  0.7398431036784295
  0.3438365483155765  0.0000000000000000  0.6287012338560667
  0.5227589742163674  0.6859615835242623  0.6385669848564195
  0.5227589742163674  0.3140384164756169  0.6385669848564195
  0.7702406886175875  0.5000000000000000  0.7678783401893805
  0.0482223632545731  0.2636013760168239  0.7565680178878915
  0.2022882654559618  0.5000000000000000  0.6586899777535065
  0.5193879261747401  0.7759317116862148  0.7415191939347878
  0.7416509947059755  0.5000000000000000  0.1482565395081048
  0.0482223632545731  0.7363986239831050  0.7565680178878915
  0.2430873343331466  0.7902441464559250  0.0607556715728074
```

0.7500590679277149	0.0000000000000000	0.3045061686495085
0.7804154841941721	0.2700387715846274	0.5462774492794887
0.2455518618052248	0.2758956225055043	0.5505527118213837
0.7415637974942640	0.7556949313572388	0.3840096960239052
0.7549829908830727	0.5000000000000000	0.4595137533788594
0.5057255936996351	0.7509219701144172	0.4674760551394488
0.2625895169449123	0.7671563608031653	0.3839729789078231
0.2592284776594437	0.5000000000000000	0.4585931544729654
0.0075328568247626	0.7530093867848090	0.4597003523829810
0.7415637974942640	0.2443050686427541	0.3840096960239052
0.4970800934372609	0.2471517026005076	0.3020464893583379
0.7562779359922163	0.0000000000000000	0.4656353180374461
0.2625895169449123	0.2328436391968347	0.3839729789078231
0.2575057691323903	0.0000000000000000	0.4700944461888670
0.0075328568247626	0.2469906132151910	0.4597003523829810
0.7526619709188470	0.7508962176493241	0.2260289762845717
0.7444373275674843	0.5000000000000000	0.3055271828233653
0.4970800934372609	0.7528482973994924	0.3020464893583379
0.2455518618052248	0.7241043774944957	0.5505527118213837
0.2508883406309660	0.5000000000000000	0.3100812659798960
0.9973212054074986	0.7472261887613293	0.3078066789543178
0.7526619709188470	0.2491037823506759	0.2260289762845717
0.5057255936996351	0.2490780298855829	0.4674760551394488
0.7163575823223461	0.2682519535913909	0.0593078508597396
0.7804154841941721	0.7299612284153726	0.5462774492794887
0.2327067267182352	0.2699630573646677	0.2258676762100036
0.2436798066725052	0.0000000000000000	0.2998065471882852
0.9973212054074986	0.2527738112386707	0.3078066789543178
0.9880720535027617	0.2524087315239528	0.1429784240981869
0.7163575823223461	0.7317480464086091	0.0593078508597396
0.2337244100012826	0.5000000000000000	0.1394192487874628
0.7400994334946116	0.0000000000000000	0.1467202860309848
0.2369831431651096	0.0000000000000000	0.1544125879313238
0.9880720535027617	0.7475912684760401	0.1429784240981869
0.0016760647707103	0.0000000000000000	0.5271722732656609
0.4894892133299883	0.0000000000000000	0.0854677592534123
0.5144467613385295	0.0000000000000000	0.5314863350985691
0.5126752435277536	0.5000000000000000	0.5285278224777182
0.5017062736044411	0.5000000000000000	0.3788018370456028
0.4943637508224136	0.0000000000000000	0.2303690042683609
0.9906510488055106	0.0000000000000000	0.0833074482265530
0.9898374505136635	0.5000000000000000	0.2307138460247432
0.5046572980242057	0.0000000000000000	0.3777511334655450
0.9952165204178117	0.0000000000000000	0.2310542246404918
0.9989191502474144	0.0000000000000000	0.3783056984861189
0.4941381916512691	0.5000000000000000	0.2311535133092822
0.9745527354423679	0.5000000000000000	0.0772324302210944
0.4916322532628247	0.5000000000000000	0.0818412328926252
0.9976588370198145	0.5000000000000000	0.3783716212476291
0.0081939384239811	0.5000000000000000	0.5227831528608421
0.7402077064613561	0.2513251566097950	0.1533056028859370
0.7547970379365080	0.7501924947355278	0.4575469902111059
0.2375628062243236	0.2522692906103218	0.1530683181950689
0.7402077064613561	0.7486748433902121	0.1533056028859370
0.2472317096460698	0.7494687611625450	0.3045635065566304
0.7471361063259501	0.7507015067222048	0.3051660279941575
0.7471361063259501	0.2492984932777952	0.3051660279941575
0.2375628062243236	0.7477307093896782	0.1530683181950689
0.2578034450729935	0.2475839906343875	0.4561228034398508
0.2472317096460698	0.2505312388374550	0.3045635065566304

```
  0.7547970379365080  0.2498075052644721  0.4575469902111059
  0.2578034450729935  0.7524160093656055  0.4561228034398508
  0.9934213211810208  0.0000000000000000  0.8849535539977272
  0.5444122337348615  0.5000000000000000  0.7099256565725559
  0.2661196315414847  0.7481258745683798  0.6257567302311238
  0.9488698605442859  0.5000000000000000  0.9048097413247972
  0.7831364064177228  0.2528142328528649  0.6257487908638042
  0.4758558721986576  0.0000000000000000  0.9021261198147611
  0.9782556246896811  0.0000000000000000  0.7187329455184475
  0.0195100552769428  0.5000000000000000  0.7246531997317452
  0.7831364064177228  0.7471857671471280  0.6257487908638042
  0.7420429215497371  0.7863884430224726  0.7963387645924428
  0.7289205879666582  0.2014604673484867  0.9842528310817151
  0.4740417002066777  0.5000000000000000  0.8977470469460274
  0.3038981275616508  0.2207783753788458  0.7876637537873774
  0.3038981275616508  0.7792216246210547  0.7876637537873774
  0.2404208070481793  0.2525796690007008  0.9853970320840241
  0.7289205879666582  0.7985395326515133  0.9842528310817151
  0.5750877359072861  0.0000000000000000  0.6937851919432887
  0.7420429215497371  0.2136115569775203  0.7963387645924428
  0.2404208070481793  0.7474203309992992  0.9853970320840241
  0.2661196315414847  0.2518741254316202  0.6257567302311238
```

Model C

```
 O Sr Ti  Y Zr
    1.0000000000000000
      7.8800000000000000    0.0000000000000000    0.0000000000000000
      0.0000000000000000    7.8800000000000000    0.0000000000000000
      0.0000000000000000    0.0000000000000000   27.3600000000000000
   O    Sr   Ti   Y    Zr
  91  12  16   2  18
Direct
  0.9125979279191654  0.7500000000000000  0.0789987798173968
  0.1370691884805950  0.7500000000000000  0.8158216125817432
  0.7447215271942014  0.0238719629867177  0.8359879131626329
  0.3443305085254522  0.4397659995445693  0.8209799534637199
  0.5359460327895746  0.2500000000000000  0.9038926410113532
  0.6764109664697671  0.7500000000000000  0.7513581481130629
  0.1328408888091417  0.7500000000000000  0.6231854741629661
  0.3756297324822242  0.4924247977586091  0.6337540950364442
  0.3756297324822242  0.0075752022412772  0.6337540950364442
  0.5909559746843200  0.2500000000000000  0.7502382366332299
  0.2972564200861996  0.5242540096614405  0.9182476154773198
  0.8898995116330539  0.0250506481809012  0.7393728689587107
  0.3591617579472199  0.9550386958442303  0.7293121954344670
  0.1588753145335033  0.2500000000000000  0.7375460021740565
  0.8511442386131733  0.9496624046946494  0.6459680289380927
  0.7447215271942014  0.4761280370131686  0.8359879131626329
  0.5036663818883227  0.7500000000000000  0.8254292663668394
  0.8898995116330539  0.4749493518190206  0.7393728689587107
  0.8191040884122529  0.4603409757905368  0.9280672331041728
  0.0458542192368299  0.2500000000000000  0.8280934577148358
  0.2972564200861996  0.9757459903385595  0.9182476154773198
  0.0419143791195111  0.2500000000000000  0.6450470005246558
  0.5891636533661426  0.7500000000000000  0.6534976956973040
  0.6392819519105970  0.2500000000000000  0.9897527162318056
  0.9935073143458411  0.7500000000000000  0.5437676644685894
  0.6522012052743165  0.2500000000000000  0.6495626494818296
  0.3443305085254522  0.0602340004553170  0.8209799534637199
  0.5683719448899538  0.7500000000000000  0.9216244804773197
```

0.8511442386131733	0.5503375953053506	0.6459680289380927
0.0789847144266744	0.2500000000000000	0.9209365773404272
0.4823273490840236	0.7500000000000000	0.0035587090376552
0.8191040884122529	0.0396590242094632	0.9280672331041728
0.2607283880841820	0.5586398791151481	0.5415091649741512
0.8108434138817344	0.6582240874616687	0.9934062332108482
0.1417276948167512	0.2500000000000000	0.5598749941000705
0.3087249270891235	0.3424224472926340	0.9916226021335888
0.3087249270891235	0.1575775527073660	0.9916226021335888
0.1508629199414031	0.7500000000000000	0.9910016884227275
0.9830828507863529	0.2500000000000000	0.0039867474135136
0.6261179066152280	0.7500000000000000	0.5613426156947483
0.5281374870945541	0.2500000000000000	0.5542769252554720
0.8263659279536668	0.4187919922288472	0.5621196007983187
0.8263659279536668	0.0812080077711599	0.5621196007983187
0.0356809566612881	0.7500000000000000	0.9101960552502177
0.2607283880841820	0.9413601208848519	0.5415091649741512
0.8108434138817344	0.8417759125383313	0.9934062332108482
0.0966659112740231	0.7500000000000000	0.7230078807352029
0.3591617579472199	0.5449613041557697	0.7293121954344670
0.1284981573675310	0.5028875372980934	0.0503686221245587
0.7083422164835014	0.2500000000000000	0.1269956145471269
0.6788609182859204	0.0001529230426485	0.1995785653161803
0.4291573409770777	0.2500000000000000	0.1978928005014851
0.9129714928028392	0.2500000000000000	0.0535952243612741
0.1775104738715712	0.7500000000000000	0.2736872012421188
0.1479956220434104	0.2500000000000000	0.1288515832140291
0.1792244452764749	0.0004726143704374	0.2012858899065790
0.9302680875922604	0.2500000000000000	0.2060549295597838
0.6268038847445325	0.4961307308544463	0.0482159778890434
0.6746828403286287	0.5002953142871931	0.4840248084051737
0.6746828403286287	0.9997046857128069	0.4840248084051737
0.9322761304877503	0.7500000000000000	0.4926214654677068
0.2243373839588330	0.2500000000000000	0.4238804492754156
0.1987718407692896	0.0004686039993373	0.4931973575687465
0.9435391582405401	0.2500000000000000	0.4773699691597031
0.6677402672185337	0.2500000000000000	0.4166886522821969
0.6845333368114908	0.7500000000000000	0.2725481407349690
0.6840954170984688	0.4999652554090375	0.3441980927643868
0.1838788018979187	0.0004939903611287	0.3483026216418779
0.4329200925913881	0.7500000000000000	0.3482408536770691
0.9364539350205661	0.2500000000000000	0.3496002958319835
0.1792244452764749	0.4995273856295626	0.2012858899065790
0.4255235177807037	0.7500000000000000	0.4749002078408324
0.4333618546883500	0.2500000000000000	0.3431829277147571
0.6840954170984688	0.0000347445909625	0.3441980927643868
0.6783038238449564	0.2500000000000000	0.2722277797950952
0.1553966789756345	0.7500000000000000	0.4205056837032615
0.1987718407692896	0.4995313960006698	0.4931973575687465
0.9335431128227327	0.7500000000000000	0.3427329208870944
0.1838788018979187	0.4995060096388713	0.3483026216418779
0.4116763659622293	0.2500000000000000	0.0784850902367040
0.1828356248215783	0.2500000000000000	0.2751119656453014
0.9302762258494397	0.7500000000000000	0.1977227132794823
0.7042240640983906	0.7500000000000000	0.4162324435023166
0.6463385564711643	0.7500000000000000	0.1276388375554518
0.6788609182859204	0.4998470769573515	0.1995785653161803
0.4294518946129386	0.7500000000000000	0.2060884190866901
0.6268038847445325	0.0038692691455537	0.0482159778890434
0.1284981573675310	0.9971124627019066	0.0503686221245587

```
  0.2091258063006549  0.7500000000000000  0.1282310966468714
  0.4479729424100611  0.2500000000000000  0.5058994965301977
  0.4152400595638568  0.7500000000000000  0.0536936823820895
  0.9138071756295432  0.9995615385596182  0.4179724630210018
  0.9138071756295432  0.5004384614403818  0.4179724630210018
  0.4415588787929678  0.4956173218752014  0.4180078283439528
  0.4415588787929678  0.0043826781247986  0.4180078283439528
  0.9264787465575566  0.9992957925645243  0.2744258819601697
  0.4261229119565826  0.5079839455003281  0.1315234192711472
  0.4271427532419168  0.9984195876263371  0.2747574764750311
  0.9255805555469507  0.0064148931036812  0.1315688017733407
  0.9264787465575566  0.5007042074354757  0.2744258819601697
  0.4261229119565826  0.9920160544996719  0.1315234192711472
  0.4271427532419168  0.5015804123736629  0.2747574764750311
  0.9255805555469507  0.4935851068963188  0.1315688017733407
  0.7140545027012521  0.2500000000000000  0.5042923384039923
  0.6432251572818117  0.2500000000000000  0.0596120768191213
  0.6537079879169312  0.7500000000000000  0.4903921354985954
  0.1721195110233964  0.2500000000000000  0.1983274248320726
  0.6749544931646625  0.2500000000000000  0.2038511820381004
  0.6857669964194528  0.7500000000000000  0.0549399204016865
  0.1443646628324302  0.7500000000000000  0.0603946670138740
  0.1744184171014993  0.2500000000000000  0.4908844467216440
  0.1875420848052798  0.2500000000000000  0.0533451554390965
  0.1761022145330600  0.7500000000000000  0.2045002769972228
  0.1807307123142508  0.2500000000000000  0.3436599155490256
  0.1900719382474847  0.7500000000000000  0.4954059782596545
  0.6755345736714702  0.2500000000000000  0.3492615987988685
  0.1717215275736024  0.7500000000000000  0.3511102029306628
  0.6772801444776277  0.7500000000000000  0.3459249981292629
  0.6731728138472874  0.7500000000000000  0.2008415517346691
  0.3806220728820335  0.2500000000000000  0.6835573242685129
  0.7859824276938738  0.7500000000000000  0.8728348994587947
  0.0667294977149453  0.5115836297747833  0.9643673474169958
  0.5784750656029090  0.4919413643424093  0.9644723516025238
  0.5784750656029090  0.0080586356575907  0.9644723516025238
  0.0667294977149453  0.9884163702252167  0.9643673474169958
  0.1416189908765872  0.5024427573562349  0.7708019012135736
  0.1416189908765872  0.9975572426436798  0.7708019012135736
  0.2946514326357530  0.2500000000000000  0.8751430221002892
  0.5719801618115667  0.5162800518560431  0.7834829944321982
  0.0486868277726131  0.0251614812257941  0.5948916098099488
  0.6255203268353853  0.5205780764308230  0.6049771847288881
  0.8561848360491524  0.2500000000000000  0.7012582328343413
  0.3016974626368452  0.7500000000000000  0.8787473259179421
  0.6255203268353853  0.9794219235691770  0.6049771847288881
  0.0486868277726131  0.4748385187742059  0.5948916098099488
  0.8513483959570962  0.7500000000000000  0.6965970888437027
  0.5719801618115667  0.9837199481439569  0.7834829944321982
  0.8411518570787493  0.2500000000000000  0.8761676489958461
  0.3257948374594335  0.7500000000000000  0.6718910187554101
```

Model D

```
 O Sr Ti  Y Zr
 1.0000000000000000
     7.8799999999999999    0.0000000000000000    0.0000000000000000
     0.0000000000000000    7.8799999999999999    0.0000000000000000
     0.0000000000000000    0.0000000000000000   27.6600000000000001
   O     Sr    Ti    Y     Zr
  87  16  12   2  18
```

```
Direct
  0.2561386581981488  0.7569804045067392  0.2054736685736260
  0.2401412983718246  0.0000000000000000  0.5431014173647311
  0.2276530388693347  0.5000000000000000  0.5935502538014105
  0.7200171085754101  0.5000000000000000  0.9801310790192564
  0.4753257079673001  0.7611588727078570  0.9110811344057074
  0.3057266775370946  0.0000000000000000  0.8142053263139163
  0.9379066253600381  0.2569931623736439  0.8329167922939351
  0.5089833894008581  0.7003451050400819  0.8135574343604567
  0.7241848910697281  0.5000000000000000  0.8709379011816905
  0.8439230641691230  0.0000000000000000  0.7467168702661677
  0.5041977139158773  0.2550175950262052  0.5779762886483365
  0.0503630841280787  0.2744467025037459  0.7356828105172207
  0.5307138399320909  0.2134714061525713  0.7231658902126709
  0.3174373837864257  0.5000000000000000  0.7358014779764311
  0.0228349372435985  0.2035948010123425  0.6374755930692964
  0.9379066253600381  0.7430068376262566  0.8329167922939351
  0.6864532450659458  0.0000000000000000  0.8227303965825618
  0.0503630841280787  0.7255532974961830  0.7356828105172207
  0.9558558863481873  0.7297382785113402  0.9218901981027476
  0.2160533333608152  0.5000000000000000  0.8292295502000864
  0.4753257079673001  0.2388411272921288  0.9110811344057074
  0.2552521584184945  0.5000000000000000  0.6474342505777086
  0.7562001500937615  0.0000000000000000  0.6554491735792841
  0.2281290734127709  0.0000000000000000  0.9132537921650822
  0.0090155174681580  0.7430060713441194  0.5466206808254601
  0.5350039990188677  0.7383675498865786  0.6314013537261555
  0.3006773899870578  0.0000000000000000  0.6309420017331695
  0.5307138399320909  0.7865285938474287  0.7231658902126709
  0.2616418912764375  0.0000000000000000  0.7240908327744933
  0.8217035901102540  0.5000000000000000  0.6575207821286287
  0.5089833894008581  0.2996548949597903  0.8135574343604567
  0.7165225482474635  0.0000000000000000  0.9174084634127214
  0.0228349372435985  0.7964051989876504  0.6374755930692964
  0.2331787413790517  0.5000000000000000  0.9162425532297080
  0.0090155174681580  0.2569939286558877  0.5466206808254601
  0.7490304840964656  0.0000000000000000  0.0055504116194056
  0.5041977139158773  0.7449824049738017  0.5779762886483365
  0.9942434840221457  0.7416451327415929  0.9749197933635448
  0.4833260976487548  0.7604633926825658  0.0049266292737851
  0.9942434840221457  0.2583548672583998  0.9749197933635448
  0.4833260976487548  0.2395366073174271  0.0049266292737851
  0.2371051477192544  0.0000000000000000  0.0050911828082893
  0.2549836463692478  0.5000000000000000  0.9707179638139264
  0.7653958331702968  0.0000000000000000  0.5613266348481575
  0.7632554040786022  0.5000000000000000  0.5642996857410898
  0.9558558863481873  0.2702617214886669  0.9218901981027476
  0.5350039990188677  0.2616324501133077  0.6314013537261555
  0.7604045617727690  0.5000000000000000  0.7554370292031177
  0.2561386581981488  0.2430195954932537  0.2054736685736260
  0.2408576363146644  0.5000000000000000  0.1400700048773894
  0.7379139598328230  0.0000000000000000  0.1307808069350759
  0.2451102850380097  0.0000000000000000  0.1287880653242656
  0.9917824448750658  0.2422215329901488  0.1395272438328216
  0.9957358249905361  0.2548656294732068  0.2726193821547722
  0.2418285326986850  0.0000000000000000  0.2753858256869938
  0.0021261957353332  0.7521224614871526  0.4236751542486755
  0.7680043710882016  0.7309202473310428  0.0622292015863521
  0.9917824448750658  0.7577784670098441  0.1395272438328216
  0.4959238085002232  0.2470507981511568  0.2788063609000548
```

0.7499421927895035	0.0000000000000000	0.2763866827319922
0.7218716541947194	0.2479324716642251	0.4877834954503716
0.2894662540680633	0.2875425623382319	0.4914843338378391
0.7608057731857658	0.7466775666004634	0.3459248756377852
0.7532499583620874	0.5000000000000000	0.4179415230959069
0.4993713740495523	0.7505200415378411	0.4115067735232791
0.7218716541947194	0.7520675283357748	0.4877834954503716
0.2360898592937429	0.7599494071968190	0.3463496449067190
0.2086259428776433	0.7174097894231923	0.0622972132905466
0.7443435465716997	0.5000000000000000	0.1369196470466217
0.7312845193250924	0.2452976503410110	0.2049555192082448
0.9957358249905361	0.7451343705268003	0.2726193821547722
0.2495955222068472	0.5000000000000000	0.2764565167551363
0.2894662540680633	0.7124574376617682	0.4914843338378391
0.4959238085002232	0.7529492018488432	0.2788063609000548
0.7415258027919622	0.5000000000000000	0.2744501949483787
0.7312845193250924	0.7547023496589891	0.2049555192082448
0.7680043710882016	0.2690797526689572	0.0622292015863521
0.0021261957353332	0.2478775385128333	0.4236751542486755
0.2360898592937429	0.2400505928031739	0.3463496449067190
0.4993713740495523	0.2494799584621590	0.4115067735232791
0.7495920706702393	0.0000000000000000	0.4163972945802051
0.7608057731857658	0.2533224333995437	0.3459248756377852
0.4915235721616018	0.2547943850864935	0.1283553432797463
0.2086259428776433	0.2825902105768147	0.0622972132905466
0.4915235721616018	0.7452056149135137	0.1283553432797463
0.2549529037987442	0.0000000000000000	0.4259648631630892
0.2448874467263676	0.5000000000000000	0.4128215564016692
0.9910527664513860	0.0000000000000000	0.0627368493929310
0.9830890802694551	0.0000000000000000	0.4909106522428244
0.4943134749465568	0.0000000000000000	0.0640248693270066
0.5115077338812551	0.0000000000000000	0.4960829621942539
0.9939246841985903	0.5000000000000000	0.4912428439518310
0.5253295161895299	0.5000000000000000	0.5034155820645531
0.4980751532774981	0.5000000000000000	0.3484953021821394
0.4990352405183860	0.5000000000000000	0.0580629026116985
0.9808115213858859	0.5000000000000000	0.0459021846931102
0.4935553084232619	0.5000000000000000	0.2030107194225020
0.0008994065709159	0.0000000000000000	0.3506326177833742
0.0014156422074351	0.5000000000000000	0.3490886579112075
0.4952981058287081	0.0000000000000000	0.3487606860082626
0.9938275993526581	0.5000000000000000	0.2035353938065895
0.4929022295950407	0.0000000000000000	0.2020472496857828
0.9963453460762962	0.0000000000000000	0.2041497455763231
0.2380904486067629	0.2555179185949724	0.1280631360350718
0.7477447330777173	0.7494864996320735	0.4218353574481293
0.7461695406492237	0.7470612425653538	0.1293222409749110
0.2467178771114486	0.7498215635299174	0.2763683254636789
0.7461695406492237	0.2529387574346391	0.1293222409749110
0.7466945336695313	0.7499504842286806	0.2758884844514952
0.7466945336695313	0.2500495157713194	0.2758884844514952
0.2380904486067629	0.7444820814050276	0.1280631360350718
0.2581162580642677	0.2542363257532837	0.4261317251091358
0.2467178771114486	0.2501784364700755	0.2763683254636789
0.7477447330777173	0.2505135003679264	0.4218353574481293
0.2581162580642677	0.7457636742467162	0.4261317251091358
0.5501123388754537	0.5000000000000000	0.6863272227612485
0.9530079996986869	0.0000000000000000	0.8742216794250340
0.2699090365119101	0.7846095679304009	0.9657871764425571
0.7093936147312996	0.7684779975959765	0.9700952137530097

```
  0.7093936147312996  0.2315220024040236  0.9700952137530097
  0.2699090365119101  0.2153904320695990  0.9657871764425571
  0.3033228287144842  0.7642766392705609  0.7663415968979668
  0.3033228287144842  0.2357233607293538  0.7663415968979668
  0.4727049222816582  0.5000000000000000  0.8648950511321439
  0.7549983092671791  0.7662000032103318  0.7809118037543756
  0.2258031860451076  0.7847005163620934  0.5842670923281957
  0.7991560169685956  0.7523988508794086  0.5888864626381133
  0.0320231919158079  0.5000000000000000  0.6974916880627364
  0.4818569470894649  0.0000000000000000  0.8736183320237371
  0.7991560169685956  0.2476011491205985  0.5888864626381133
  0.7549983092671791  0.2337999967896611  0.7809118037543756
  0.0068831924736623  0.0000000000000000  0.6861629843720094
  0.2258031860451076  0.2152994836379066  0.5842670923281957
  0.9886363450193904  0.5000000000000000  0.8685484940282099
  0.5078674389494541  0.0000000000000000  0.6751948279667147
```

Model E

```
 O Sr Ti  Y Zr
    1.0000000000000000
      7.8800000000000000    0.0000000000000000    0.0000000000000000
      0.0000000000000000    7.8800000000000000    0.0000000000000000
      0.0000000000000000    0.0000000000000000   27.1800000000000000
    O    Sr   Ti   Y    Zr
   83  12  16   2  18
Direct
  0.9571785800717295  0.7500000000000000  0.1003570399251400
  0.9253678890736352  0.2500000000000000  0.6538109634484867
  0.7379415469629241  0.0216055221132466  0.7447597222710679
  0.5250523122575520  0.2500000000000000  0.8090101087695248
  0.1593018148584630  0.0063146772320299  0.6375501861626667
  0.7379415469629241  0.4783944778866823  0.7447597222710679
  0.1593018148584630  0.4936853227678565  0.6375501861626667
  0.7562542674170646  0.7500000000000000  0.0171192328534243
  0.1875226754548737  0.5346444878158515  0.7318838752892688
  0.8287728714961314  0.4657766401873928  0.8438612729308475
  0.6738702829700666  0.9901787573279338  0.6485266138799721
  0.4172809559416938  0.7500000000000000  0.6673691353356537
  0.6738702829700666  0.5098212426720804  0.6485266138799721
  0.6577034827056585  0.7500000000000000  0.9376640472123130
  0.4660484808882474  0.2500000000000000  0.6962358894954990
  0.9251047509626491  0.7500000000000000  0.7280695066122598
  0.9191367474401864  0.7500000000000000  0.6322894297248457
  0.9714368082451091  0.2500000000000000  0.7481946574164284
  0.5565588857298558  0.7500000000000000  0.7604759035813328
  0.5026737610552651  0.2500000000000000  0.5040949396878105
  0.4220879942672730  0.9668872258713622  0.9671795968825694
  0.3910125966988903  0.9820776109973725  0.8609553152768392
  0.6305313724629744  0.7500000000000000  0.8486346388810651
  0.0222713953519907  0.7500000000000000  0.8179135105010911
  0.9202676358594454  0.4988593424208794  0.9520188591526214
  0.7009180930287892  0.2500000000000000  0.0112297674650406
  0.1516002849735029  0.7500000000000000  0.0148928530732153
  0.1883212939999116  0.2500000000000000  0.0127290325083162
  0.5910579612357910  0.7500000000000000  0.5845059775227691
  0.7244245206595585  0.2500000000000000  0.5885713084471855
  0.2663408088897851  0.7500000000000000  0.5909331830465350
  0.3698738996407530  0.2500000000000000  0.5957247177596724
  0.4220879942672730  0.5331127741286378  0.9671795968825694
  0.8287728714961314  0.0342233598125006  0.8438612729308475
```

0.1197336413244918	0.2500000000000000	0.8288712663202352
0.3910125966988903	0.5179223890024997	0.8609553152768392
0.6664292340746227	0.2500000000000000	0.9180910569353538
0.1708800946304394	0.7500000000000000	0.9234790703547425
0.9202676358594454	0.0011406575791347	0.9520188591526214
0.1551067667641027	0.2500000000000000	0.9239938674191137
0.9664788320758380	0.7500000000000000	0.2286055563982217
0.1875226754548737	0.9653555121841485	0.7318838752892688
0.6947011606917405	0.7500000000000000	0.1583548077641225
0.9664636211723556	0.2500000000000000	0.2344161105391169
0.7743395483610648	0.5035970780644468	0.5321389397064052
0.2281779373267087	0.0020756384055646	0.3779542340827532
0.7743395483610648	0.9964029219355532	0.5321389397064052
0.0008289646269114	0.7500000000000000	0.5240013856648100
0.1978278369496517	0.2500000000000000	0.4609745498700930
0.2922556394923045	0.9986009843354093	0.5335758878577773
0.0258428518678461	0.2500000000000000	0.5565124563635889
0.7855201222452570	0.2500000000000000	0.4518580225807636
0.7226412644791498	0.7500000000000000	0.3026299926756426
0.7288018552178457	0.4984115276664317	0.3773774806212253
0.4786407016638425	0.7500000000000000	0.3767598131539948
0.4539561165761484	0.7500000000000000	0.0862110388524607
0.2235716103103833	0.7500000000000000	0.3032212733796928
0.2423503945389019	0.2500000000000000	0.3043088828875520
0.2281779373267087	0.4979243615944354	0.3779542340827532
0.9785488143325817	0.7500000000000000	0.3714877816863697
0.2922556394923045	0.5013990156645907	0.5335758878577773
0.2140941517029208	0.7500000000000000	0.4482134056518933
0.7073594216971760	0.2500000000000000	0.3047849611028124
0.7288018552178457	0.0015884723335683	0.3773774806212253
0.4796288765325502	0.2500000000000000	0.3839492560557090
0.4941366588437063	0.7500000000000000	0.4978682077952754
0.2150587619010911	0.0007011060629099	0.2299479520381668
0.7581521796227111	0.7500000000000000	0.4475065186941729
0.2046061514034637	0.2500000000000000	0.1574203665358422
0.9536603810633131	0.2500000000000000	0.0913399375575112
0.2011941648001132	0.4995606337491731	0.0713780284364241
0.7170875412307400	0.4989472091242483	0.2299900285677903
0.6998310224653396	0.9994707813726211	0.0719575701376219
0.2011941648001132	0.0004393662508269	0.0713780284364241
0.4658429516027169	0.7500000000000000	0.2331589147397309
0.2150587619010911	0.4992988939370900	0.2299479520381668
0.2256369618443668	0.7500000000000000	0.1573175609297258
0.9813815869372536	0.2500000000000000	0.3700094579382335
0.6998310224653396	0.5005292186273789	0.0719575701376219
0.7155114501885429	0.2500000000000000	0.1576833289054627
0.7170875412307400	0.0010527908757517	0.2299900285677903
0.4659552147172865	0.2500000000000000	0.2283929884100380
0.4539380544514339	0.2500000000000000	0.0923688269376302
0.9757145714657189	0.5063696154819459	0.4469788749848764
0.4626770940005234	0.5003383359593663	0.4446032987439266
0.9737303636946807	0.0025351201760974	0.1568532745296604
0.9757145714657189	0.9936303845180541	0.4469788749848764
0.9680359119349050	0.9963594506306871	0.3011358275023504
0.9680359119349050	0.5036405493693129	0.3011358275023504
0.9737303636946807	0.4974648798238955	0.1568532745296604
0.4747275290937978	0.9959824903674269	0.3012321996325005
0.4735509879070384	0.5018034389314292	0.1537764805431944
0.4626770940005234	0.9996616640406266	0.4446032987439266
0.4735509879070384	0.9981965610685708	0.1537764805431944

```
  0.4747275290937978  0.5040175096325731  0.3012321996325005
  0.2278920488554396  0.7500000000000000  0.5215769822459189
  0.7279591032431113  0.2500000000000000  0.0805682395383497
  0.7049467683677305  0.7500000000000000  0.5242180454637584
  0.2253672396340584  0.2500000000000000  0.2269669923225695
  0.7225423516126540  0.2500000000000000  0.2273178200255012
  0.7327852655664543  0.7500000000000000  0.0853010107444214
  0.2248107748099345  0.7500000000000000  0.0822437263268856
  0.2488015612302306  0.2500000000000000  0.5294808537971458
  0.2264176741425348  0.2500000000000000  0.0815009824323785
  0.2262133109911000  0.7500000000000000  0.2280828321583891
  0.2187530156598199  0.2500000000000000  0.3716209708983558
  0.7216434860643133  0.2500000000000000  0.3742137667006557
  0.2182141083027174  0.7500000000000000  0.3740769952350439
  0.7225263700006866  0.7500000000000000  0.3779340461878742
  0.7190557757709332  0.2500000000000000  0.5193281348118463
  0.7231747914022418  0.7500000000000000  0.2299618168467906
  0.9045650224738518  0.7500000000000000  0.8930376116905779
  0.1891776822150202  0.2500000000000000  0.6940424047376452
  0.1434610862809780  0.7500000000000000  0.6793532508418465
  0.1724007456870137  0.5025881957816249  0.9856429617132931
  0.6805409382301632  0.5109839429974350  0.9876021518194316
  0.6805409382301632  0.9890160570025650  0.9876021518194316
  0.1724007456870137  0.9974118042183751  0.9856429617132931
  0.9871455250556768  0.5192594096423312  0.7760272197655947
  0.9871455250556768  0.9807405903575764  0.7760272197655947
  0.5891614326887605  0.5265277745068715  0.8084173881710380
  0.8962321267885206  0.0365470502251154  0.6042980320290520
  0.4069780381296800  0.5259229858650368  0.6074965188974624
  0.7127976615586855  0.2500000000000000  0.7038982411578582
  0.4224342420816223  0.7500000000000000  0.9039840285430967
  0.4069780381296800  0.9740770141349632  0.6074965188974624
  0.8962321267885206  0.4634529497748917  0.6042980320290520
  0.5891614326887605  0.9734722254931285  0.8084173881710380
  0.6783803367384691  0.7500000000000000  0.6886954219895044
  0.8951337538800616  0.2500000000000000  0.8907984639786634
  0.3301651811204790  0.2500000000000000  0.8665452437148433
```

Model F

```
 O Sr Ti  Y Zr
    1.0000000000000000
     7.8800000000000000    0.0000000000000000    0.0000000000000000
     0.0000000000000000    7.8800000000000000    0.0000000000000000
     0.0000000000000000    0.0000000000000000   26.6100000000000000
   O    Sr   Ti   Y    Zr
  79  16  12   2  18
Direct
  0.2722659226066608  0.7484118672887355  0.2036104526150573
  0.0091212259550776  0.2714519644757168  0.9245377948145098
  0.4109881485300206  0.2464714049081883  0.8488738024373057
  0.4742409004649840  0.5000000000000000  0.9130121051939437
  0.5014836534202876  0.2364559138719911  0.9716155733493466
  0.9130319897577691  0.0000000000000000  0.7401796527481710
  0.0486955841759493  0.7028044379243238  0.7298621184084420
  0.4952311415749904  0.7514450234402819  0.6333624892771955
  0.2842816649942595  0.0000000000000000  0.9150483505843033
  0.9612550256995064  0.0000000000000000  0.9692371270455583
  0.9541206694906341  0.7609727328320134  0.8289624615786403
  0.9945606533921827  0.2470985225622072  0.6337735336858765
  0.7404281106303693  0.0000000000000000  0.6618644009292867
```

0.9945606533921827	0.7529014774377999	0.6337735336858765
0.7063982477294547	0.0000000000000000	0.9094128975760902
0.7554688577362821	0.5000000000000000	0.6253008420698062
0.2551903772479335	0.0000000000000000	0.7122104981289794
0.5123202153441955	0.7649803939497004	0.7365034493005392
0.2473428830719731	0.0000000000000000	0.6159513584260849
0.7326091783702107	0.5000000000000000	0.8303392127337585
0.1927967075044572	0.5000000000000000	0.8760977856427762
0.0486955841759493	0.2971955620755979	0.7298621184084420
0.2486823026664955	0.5000000000000000	0.6622683472940702
0.5123202153441955	0.2350196060503067	0.7365034493005392
0.3186958397776316	0.5000000000000000	0.7759020902774468
0.6778520213209028	0.0000000000000000	0.8042769986014268
0.2542679546854564	0.0000000000000000	0.8086638102852948
0.0091212259550776	0.7285480355242974	0.9245377948145098
0.4109881485300206	0.7535285950916908	0.8488738024373057
0.7947229916699122	0.5000000000000000	0.9548998551640461
0.2399974823285904	0.5000000000000000	0.9778284933838659
0.9070375695513494	0.0000000000000000	0.5804585281677639
0.0826223852387226	0.5000000000000000	0.5797321170897083
0.5803507419197775	0.0000000000000000	0.5789695798574378
0.4101188651094633	0.5000000000000000	0.5747366166953088
0.5014836534202876	0.7635440861280017	0.9716155733493466
0.9541206694906341	0.2390272671678801	0.8289624615786403
0.7040819619753773	0.0000000000000000	0.0148235844124163
0.7788953194049079	0.5000000000000000	0.7285313457755234
0.4952311415749904	0.2485549765596045	0.6333624892771955
0.2722659226066608	0.2515881327112715	0.2036104526150573
0.2444396120184318	0.5000000000000000	0.1264758528860660
0.7536383011376230	0.0000000000000000	0.1248818788681642
0.2564978961946692	0.0000000000000000	0.1294928015566321
0.0000320001106169	0.2450530949611706	0.1336402907446971
0.0000861573461399	0.2503883397137702	0.2745429794763119
0.2494525986147066	0.0000000000000000	0.2805000654552216
0.5056253237621737	0.2572441195682632	0.1187453375089572
0.7909731450289610	0.7107618428900421	0.0481056769891256
0.0000320001106169	0.7549469050388293	0.1336402907446971
0.5003266480095228	0.2499028420809404	0.2845396023105537
0.7508508671191190	0.0000000000000000	0.2782232733157692
0.7456703188632601	0.2726907526914710	0.5194749149782171
0.2485086382421971	0.2313687881479035	0.5171726845365834
0.7603140189685283	0.7597630897936486	0.3557403452075894
0.7506741819087360	0.5000000000000000	0.4272734980829445
0.4994675059632794	0.7502702784413006	0.4295525923467078
0.7456703188632601	0.7273092473085362	0.5194749149782171
0.2385961246595446	0.7426284308006326	0.3561845781182842
0.2178906846869727	0.7633376892624797	0.0532307977172467
0.7566974063123514	0.5000000000000000	0.1318454490899433
0.7300457442054423	0.2476453850261928	0.2030202945571130
0.0000861573461399	0.7496116602862297	0.2745429794763119
0.2504781261416726	0.5000000000000000	0.2790624691098262
0.2485086382421971	0.7686312118520964	0.5171726845365834
0.5003266480095228	0.7500971579190454	0.2845396023105537
0.7496278325971750	0.5000000000000000	0.2798227370629540
0.2178906846869727	0.2366623107375132	0.0532307977172467
0.7909731450289610	0.2892381571099437	0.0481056769891256
0.7300457442054423	0.7523546149738071	0.2030202945571130
0.2482646544103579	0.0000000000000000	0.4280349488041735
0.2385961246595446	0.2573715691993960	0.3561845781182842
0.4994675059632794	0.2497297215587136	0.4295525923467078

0.7488732535549294	0.0000000000000000	0.4349932432674751
0.7603140189685283	0.2402369102063585	0.3557403452075894
0.9982094568897345	0.7510101004677855	0.4336880167288988
0.5056253237621737	0.7427558804317368	0.1187453375089572
0.9982094568897345	0.2489898995322144	0.4336880167288988
0.2479652904077372	0.5000000000000000	0.4337983811564145
0.0106166106897804	0.0000000000000000	0.4977723445319157
0.0280327376507343	0.5000000000000000	0.0426579113390410
0.5002178980585227	0.5000000000000000	0.1925161540362254
0.0026923035299833	0.0000000000000000	0.3457978903909663
0.9964206385714600	0.0000000000000000	0.1974985734867697
0.4986133872790391	0.0000000000000000	0.3478534963232607
0.9996268257614307	0.5000000000000000	0.1962801843313701
0.9912812739498492	0.0000000000000000	0.0600542605780779
0.9985613577690133	0.5000000000000000	0.3455883829464166
0.4994545227733944	0.5000000000000000	0.3478278563708794
0.5100819920476547	0.5000000000000000	0.4937835328568477
0.9881181599523762	0.5000000000000000	0.4964166166467082
0.4877985013461560	0.0000000000000000	0.4958296706067253
0.4218920142727894	0.0000000000000000	0.0323141264131455
0.5093287792447417	0.5000000000000000	0.0290281707404454
0.5043768282950273	0.0000000000000000	0.1907526122693401
0.2468089616191754	0.2514791871571050	0.4243759257568274
0.7518832847243362	0.7526938389691757	0.4235033435058856
0.2452839624683720	0.2510533881801192	0.1200664222483800
0.7471020579846811	0.7428894378703194	0.1173516006502719
0.2539902488452647	0.7493491749971963	0.2716451638643431
0.7471020579846811	0.2571105621296805	0.1173516006502719
0.7463516718952548	0.7510021907006050	0.2711163526568142
0.7463516718952548	0.2489978092993950	0.2711163526568142
0.2452839624683720	0.7489466118198951	0.1200664222483800
0.2539902488452647	0.2506508250027895	0.2716451638643431
0.7518832847243362	0.2473061610308242	0.4235033435058856
0.2468089616191754	0.7485208128429020	0.4243759257568274
0.0092166667160219	0.0000000000000000	0.8806969223537916
0.5284497845255324	0.5000000000000000	0.6815466099183836
0.4767536035815810	0.0000000000000000	0.6634399540797280
0.2912589116859408	0.6993702022719648	0.9279872543919547
0.7705710127436713	0.7990993776953289	0.9689668552149184
0.7705710127436713	0.2009006223046782	0.9689668552149184
0.2912589116859408	0.3006297977280495	0.9279872543919547
0.2709228450916342	0.7759955801204163	0.7655126986933799
0.2709228450916342	0.2240044198794984	0.7655126986933799
0.8153482986409119	0.7697180565375706	0.7677863406923962
0.2526080296917481	0.7029000524837120	0.5916596899577128
0.7439335181238341	0.7862941986008828	0.5932652271891511
0.9866973223845480	0.5000000000000000	0.6720106544856717
0.4990778156680250	0.0000000000000000	0.8584107394560495
0.7439335181238341	0.2137058013991172	0.5932652271891511
0.8153482986409119	0.2302819434624293	0.7677863406923962
0.2526080296917481	0.2970999475162880	0.5916596899577128
0.0180970212804037	0.0000000000000000	0.6642622888683678
0.9337705781169299	0.5000000000000000	0.8847134904139464
0.4804219488254906	0.5000000000000000	0.8378145258521305
